# Continuously Improved Photocatalytic Performance of Zn_2_SnO_4_/SnO_2_/Cu_2_O Composites by Structural Modulation and Band Alignment Modification

**DOI:** 10.3390/nano9101390

**Published:** 2019-09-28

**Authors:** Tiekun Jia, Junchao An, Dongsheng Yu, Jili Li, Fang Fu, Kun Wang, Weimin Wang

**Affiliations:** 1 School of Materials Science and Engineering, Luoyang Institute of Science and Technology, Wangcheng Road 90#, Luoyang 471023, China; dongsh_yu@163.com (D.Y.); lijili328@126.com (J.L.); fufang1@126.com (F.F.); wk13007581560@163.com (K.W.); 2 State Key Lab of Materials Synthesis and Processing, Wuhan University of Technology, Wuhan 430070, China; wangwm@hotmail.com

**Keywords:** Zn_2_SnO_4_/SnO_2_, Cu_2_O decoration, structural modulation, band alignment, photodegradation mechanism

## Abstract

Improving the photocatalytic performance of multi-component photocatalysts through structural modulation and band alignment engineering has attracted great interest in the context of solar energy utilization and conversion. In our work, Zn_2_SnO_4_/SnO_2_ hierarchical architectures comprising nanorod building block assemblies were first achieved via a facile solvothermal synthesis route with lysine and ethylenediamine (EDA) as directing agents, and then chemically etched in NaOH solution to enlarge the surface area and augment active sites. The etched Zn_2_SnO_4_/SnO_2_ hierarchical architectures were further decorated by Cu_2_O nanoparticles though an in situ chemical deposition method based on band alignment engineering. In comparison with unetched Zn_2_SnO_4_/SnO_2_, the specific surface area of Zn_2_SnO_4_/SnO_2_/Cu_2_O hierarchical architectures became larger, and the responsive region and absorbance intensity became wider and higher in the whole visible-light range. Zn_2_SnO_4_/SnO_2_/Cu_2_O hybrid photocatalysts presented enormously improved visible-light photocatalytic behaviour for Rhodamine B (RhB) decomposition. The enhancement of photocatalytic behaviour was dominantly attributed to the synergy effect of the larger specific surface area, higher light absorption capacity, and more effective photo-induced charge carrier separation and migration. A proposed mechanism for the enormously promoted photocatalytic behaviour is brought forth on the basis of the energy-band structure combined with experimental results.

## 1. Introduction

As one of the widely investigated ternary oxide materials in the application of photodegrading pollutants [1,2,3,4,5,6], Zn_2_SnO_4_ (ZSO) has the prominent advantages of relatively high electron mobility and long-term chemical stability. However, limited to its wide bandgap and unfavorable recombination rate, the photocatalytic efficiency of pure ZSO is not satisfied. Thus, researchers have taken some approaches, for instance, ion doping [2], morphological modulation [3,4,5,6], and coupling composites [7,8,9,10,11,12,13,14,15,16], to improve the photocatalytic behavior of ZSO based photocatalyst. Among those strategies mentioned above, relevant reports on doping in ZSO are very limited, probably because the concentration of dopants is not easily controlled and the impurity level can act not only as a platform for electron transition, but also serve as a recombination center. Further, we all know that it is very difficult for a single component photocatalyst to overcome the intrinsic drawbacks of a low quantum rate and high recombination rate only by morphological modulation. In this regard, constructing composites is one of the most facile and efficient solutions, because the construction of coupling ZSO with other matchable photocatalysts is beneficial for prolonging the lifespan of photo-generated charge carriers and accelerating charge carrier separation [14,15,16,17].

As previously reported [7,8,9,13,14,15], SnO_2_ is available to construct ZSO/SnO_2_ composites displaying the improvement of photodegradation rate. For instance, Chen et al. [7] took a one-pot hydrothermal synthesis method to fabricate SnO_x_/Zn_2_SnO_4_ composites, which exhibited a higher and more stable photocatalytic performance toward the degradation of MO and gaseous C_6_H_6_ under UV light irradiation. Junploy et al. [8] reported an approach involving co-precipitation combined with calcination for the synthesis of Zn_2_SnO_4_/SnO_2_, and the results showed that the as-obtained Zn_2_SnO_4_/SnO_2_ displayed improved photocatalytic efficiency toward the degradation of MB under UV light illumination, as compared to Zn_2_SnO_4_ and SnO_2_. Most previously reported studies were focused on the improvement of UV-light photocatalytic behavior over Zn_2_SnO_4_/SnO_2_ photocatalysts, while the relevant studies on visible light photocatalytic activity were relatively scarce, probably because both Zn_2_SnO_4_ and SnO_2_ belong to the group of wide band-gap photocatalysts. Generally, the photocatalytic performance in visible-light region over Zn_2_SnO_4_/SnO_2_ coupling composites is conceivably triggered by defect states originating from oxygen vacancy or interstitial ions. Han et al. [13] fabricated double-shell Zn_2_SnO_4_/SnO_2_ microboxes with superior photocatalytic performance under sun-light illumination. Zhang et al. [14] designed a reduced method to fabricate surface-disordered Zn_2_SnO_4_/SnO_2_ hybrid nanocomposites, which exhibited improved sun-light driven degradation performance of organic pollutant owing to the introduction of oxygen vacancy and amorphous shells. However, the promotion of light absorption capacity is limited only by the introduction of defects. It is desirable to indraught narrowed band photocatalyst for further boosting of the light adsorption of the Zn_2_SnO_4_/SnO_2_ hybrid. Ho et al. [15] prepared a novel visible-light-response photocatalyst Zn_2_SnO_4_-SnO_2_/graphene (rGO) with improved photo-reactivity, and the results showed that rGO performed as an electron mediator to favor the high photocatalytic efficiency toward acetone and NO oxidation. Despite the previous achievements mentioned above, it is still a challenge to achieve a novel, multi-component Zn_2_SnO_4_-SnO_2_-based photocatalyst by simultaneously extending the light absorption range and increasing the spatial separation of photogenerated charge carriers.

Cu_2_O is regarded as a promising candidate for visible-light photocatalyst owing to its suitable band-gap (2.1–2.4 eV), amazing optical property, low cost, non-toxicity, and environmental acceptability [18,19,20,21]. However, pure Cu_2_O has a short charge diffusion length (20–100 nm), resulting in a high recombination rate of photoinduced electron-hole pairs and quite low quantum efficiency [22]. For this purpose, as an efficient co-catalyst, Cu_2_O could be added into some photocatalyst systems, e.g., ZnO [21,23], TiO_2_ [24,25], CdS [26], ZnWO_4_ [27], WO_3_ [19,28], BiVO_4_ [29], BiOBr [30], in order to broaden light absorption scope, facilitate charge carriers transfer and separation, and then boost up the photocatalytic performance. Inspired by previously reported achievements, we developed a novel “directing growth-etching treatment-coupling composites” route to prepare Zn_2_SnO_4_/SnO_2_/Cu_2_O hybrid photocatalyst based on structural modulation and band alignment modification in this work. After the overall modification mentioned above, the as-obtained Zn_2_SnO_4_/SnO_2_/Cu_2_O hybrids were simultaneously endowed with a higher specific surface area, wider spectral absorption range, and more efficient spatial separation of photogenerated charge carriers, giving rise to an enormous improvement of photodegradation efficiency for the removal of RhB. To our certain knowledge, this is the first report on the fabrication and insight into the mechanism of enhanced photocatalytic behavior of Zn_2_SnO_4_/SnO_2_/Cu_2_O hybrids.

## 2. Materials and Methods

### 2.1. Materials

Sodium stannate tetrahydrate (Na_2_SnO_3_·4H_2_O), zinc acetate dehydrate (Zn(CH_3_COO)_2_·2H_2_O), EDTA disodium (EDTA-2Na), sodium hydroxide (NaOH), lysine, copper nitrate penthydrate (Cu(NO_3_)_2_·5H_2_O), ascorbic acid (AA), and rhodamine B (RhB) were all obtained from Sinopharm Chemical Reagent CO., Ltd. (Shanghai, China). Ethanol and ethylenediamine (EDA) were purchased from Fuyu Fine Chemical Reagent CO., Ltd. (Tianjin, China). All the chemicals used in this work were of analytical grade without further purification.

### 2.2. Synthesis of Pristine Zn_2_SnO_4_/SnO_2_ Hierarchical Architectures (P-ZSO/SnO_2_)

P-ZSO/SnO_2_ was fabricated through a facile solvothermal synthesis procedure. Briefly, 1 mmol of Zn(CH_3_COO)_2_·2H_2_O and 2.5 mmol of Na_2_SnO_3_·4H_2_O were respectively put into the mixed solvents containing 27 mL deionized water, 8 mL EDA and 5 mL anhydrous alcohol under intense stirring to form a transparent solution. For simplicity, the solution containing Zn^2+^ was denoted as solution A, while the solution containing Sn^4+^ as solution B. The solution A was slowly dripped into solution B under vigorous stirring. After that, 0.2 mmol of lysine was added to the above mixture, which was continuously kept stirring for another 30 min. The obtained suspension was transferred into a 100 mL Teflon-lined autoclave, and maintained at 190 °C for 20 h. After being naturally cooled to room temperature (the cooling time is about 7 h), the white precipitate was centrifuged and washed with deionized water and anhydrous alcohol repeatedly. The resulting precipitate was dried at 70 °C in an electric drying oven overnight, and then grinded into powder. The as-obtained product was named P-ZSO/SnO_2_. For comparison, Zn_2_SnO_4_/SnO_2_ microspheres (M-ZSO) were prepared via a facile route, and the detailed procedure was presented in the supporting information.

### 2.3. Synthesis of Etching Zn_2_SnO_4_/SnO_2_ Hierarchical Architectures (ET-ZSO/SnO_2_)

First, 0.5 g of P-ZSO/SnO_2_ was added to a 60 mL sodium hydroxide (5 M) solution with constant stirring for 20 min. Then, the suspension mentioned above was transferred into a 100 mL Teflon-lined autoclave and kept at 60 °C for 1 h. The subsequent washing and drying process was similar to that of P-Zn_2_SnO_4_/SnO_2_. The as-obtained product was denoted as ET-ZSO/SnO_2_.

### 2.4. Synthesis of Zn_2_SnO_4_/SnO_2_/Cu_2_O Hierarchical Architectures

Typically, 0.02 mmol EDTA-2Na, 0.02 mmol Cu(NO_3_)_2_·5H_2_O and a specified amount of ET-ZSO/SnO_2_ were put into 50 mL deionized water to form a suspension, and sonicated for 30 min. 0.05 mmol NaOH and 0.1 mmol AA were respectively dissolved into 20 mL deionized water under constant stirring. The NaOH-solution was added dropwise into the suspension, and then followed by the sluggish drip of the AA-solution with vigorous stirring for another 40 min. The above mixture was stirred for 240 min at room temperature. The resulting product was treated by centrifuging and washing with deionized (D.I.) water and anhydrous alcohol to eliminate the impurity, followed by drying at 120 °C in a vacuum oven overnight. In this manner, coupling composites of 0.5, 1, 1.5, and 2% Cu_2_O loaded onto ET-ZSO/SnO_2_ were prepared and denoted as ZSC0.5, ZSC1, ZSC1.5 and ZSC2, respectively.

### 2.5. Characterizations

The crystal structure analysis of P-ZSO/SnO_2_, ET-ZSO/SnO_2_, and ZSC hybrids were performed by using a D8 Advance X–ray diffractometer (Bruker, Billerica, MA, USA) with a copper radiation (λ = 0.15406 nm). An ESCALAB 250Xi spectrometer (Thermo Fisher Scientific, New York, NY, USA) equipped with a monochromatic Al-Kα radiation was employed to examine the surface component of P-ZSO/SnO_2_ and ZSC hybrids. Morphological and micro-structural investigations of P-ZSO/SnO_2_ and ZSC hybrids were achieved on a Hitachi-S-4800 field emission scanning electron microscope (Hitachi, Toyko, Japan) as well as JEM-2100F transmission electron microscope (JEOL Ltd., Tokyo, Japan). The Brunauer–Emmett–Teller (BET) specific surface area of P-ZSO/SnO_2_ and ZSC hybrids was determined on the basis of multi-point estimation using a NOVA-2000e sorption analyzer. UV-DRS of P-ZSO/SnO_2_, ET-ZSO/SnO_2_ and ZSC hybrids were collected on a TU 1901 UV–vis spectrophotometer (Puxi, Beijing, China), using BaSO_4_ as a reference. Photoluminescence (PL) spectra excitated with a wavelength of 320 nm were achieved on a LS55 fluorescence spectrophotometer (PE, Waltham, MA, USA).

### 2.6. Photocatalytic Experiments

Commonly used RhB dye in the textile industry was taken as a model pollutant in our work. The procedure of RhB photodegradation tests were performed in a homemade apparatus, which was reported in our previous studies [10,11,12,31,32]. Briefly, 50 mg of photocatalyst was coated on the bottom surface of the culture dish (9 cm) to form a thin film, followed by dispersing into 40 mL RhB aqueous solution (1.0 × 10^−5^ M). Two daylight lamps (60 W, λ ≥ 420 nm) were designated as the visible light source for triggering the photodegration reaction of RhB. After a specified interval of the simulant sun-light illumination, the suspension was sampled and the concentration of the reacted RhB aqueous solution was measured on a TU 1901 UV–vis spectrophotometer with the wavelength ranging from 300 nm to 700 nm.

### 2.7. Photocurrent Measurements

The photo-electrochemical measurements of P-ZSO/SnO_2_, Cu_2_O, and ZSC1.5 samples were carried out on a three-electrode CHI660E electrochemical workstation (Chenhua Instruments Co., Shanghai, China), which contained the counter electrode (Pt wire), reference electrode (saturated Ag/AgCl), and working electrode (photocatalysts). The details for the photo-electrochemical measurements were presented in our previously reported study [31,32].

## 3. Results and Discussion

**Figure 1:**
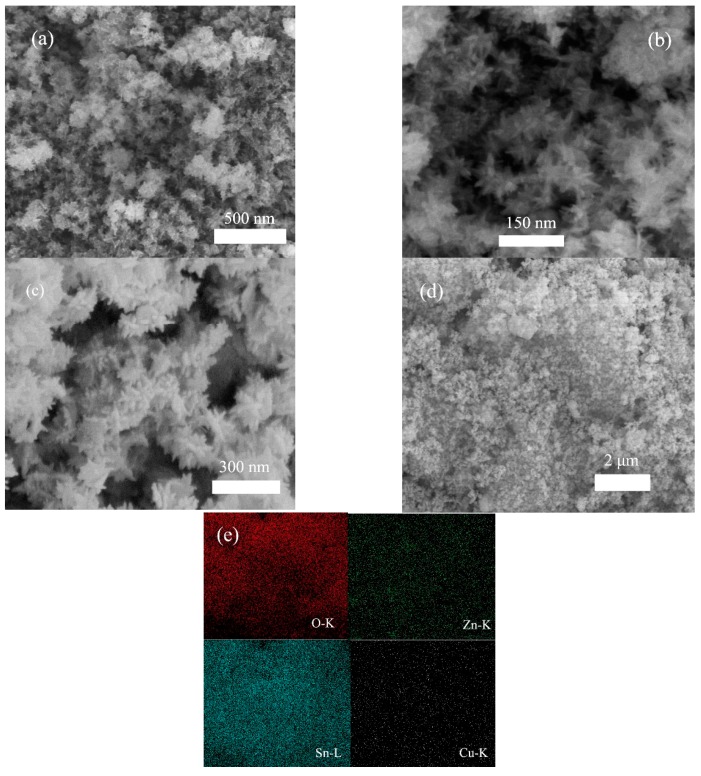
(**a**,**b**) Low-magnification and high-magnification FESEM images of P-ZSO/SnO_2_, (**c**) FESEM image of the ZSC1.5 sample, (**d**,**e**) SEM image and its corresponding EDS mapping of the ZSC1.5 sample.

Considering that 3D hierarchical architectures composed of one dimensional (1D) nanobuilding blocks, with desired size, shape and composition, play substantial roles in determining the photo-activity of photocatalysts, the morphological structure and composition of the as-obtained products were characterized by field emission scanning electron microscope (FESEM) in combination with EDS analysis, and TEM. From the FESEM image with low-magnification (Figure 1a), P-ZSO/SnO_2_ was made up of massive flower-like architectures. High-magnification FESEM micrograph (Figure 1b) revealed clearly morphological structure that 3D flower-like architectures of P-ZSO/SnO_2_ were comprised of a large number of closely interconnected 1D nanorod building blocks with small size. Comparing Figure 1b with Figure 1c, we can easily find that the ZSC1.5 sample almost inherited the hierarchical morphology of the corresponding P-ZSO/SnO_2_ product, signifying that the etching process in combination with chemical deposition procedure used in this experiment only exerted a limited effect on the morphological structure. Such a special morphological structure will be beneficial for achieving high photo-activity [33,34,35]. The result of energy dispersive spectrometer (EDS) mapping (Figure 1e) perfectly authenticated the coexistence and homogeneous distribution of Zn, Sn, O, and Cu elements throughout the ZSC1.5 sample. Additionally, as is seen from Supplementary Figure S1, the M-ZSO sample was composed of microsphere-shaped particles with the size ranging from 1 to 2 μm.

**Figure 2:**
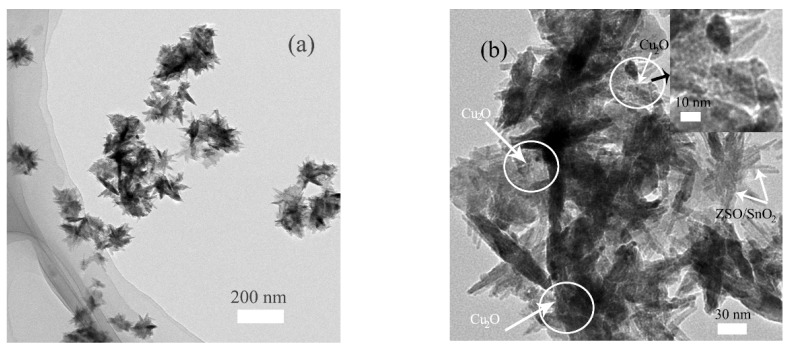
(**a**) Low-magnification TEM image, and (**b**) high-magnification TEM image as well as partially magnified image in the inset of the ZSC1.5 sample.

A deep insight into detailed structural features of the ZSC1.5 sample was provided by TEM observation, as shown in Figure 2a,b. Obviously, the obtained ZSC1.5 sample exhibited 3D flower-like architectures with a mean size of 100–200 nm, built up from plenty of nanorods with an average diameter of approximately 10–20 nm. A close-view of a single unit was revealed from Figure 2b that oriented nanorods (referring to ZSO and SnO_2_) were grown together with the common part in the middle and congregated with each other to shape into flower-like architectures. Noticeably, fine discrete Cu_2_O nanoparticles were tightly anchored on the surface of ZSO and SnO_2_ nanorods to establish an intimate contact along the interface between ZSO, SnO_2_ and Cu_2_O, which would effectively accelerate the interfacial charge migration and separation.

**Figure 3:**
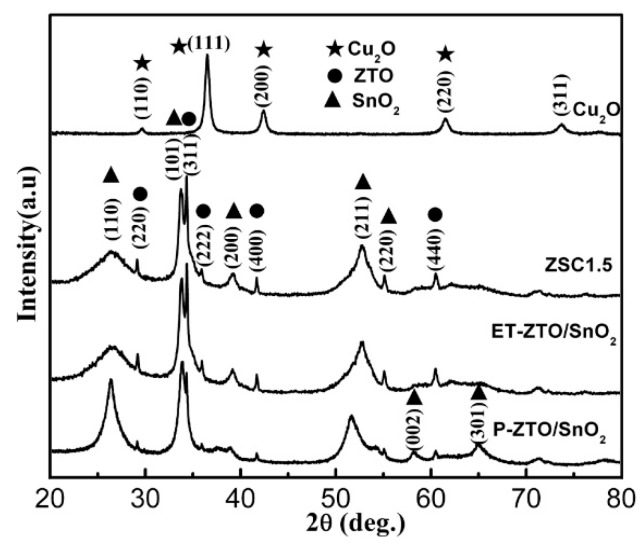
XRD patterns of P-ZSO/SnO_2_, ET-ZSO/SnO_2_, ZSC1.5, and Cu_2_O samples.

Figure 3 depicts the XRD patterns of P-ZSO/SnO_2_, ET-ZSO/SnO_2_, ZSC1.5, and Cu_2_O samples. Clearly, only two crystalline phases of ZSO and SnO_2_ can be perfectly identified in P-ZSO/SnO_2_, well indexed to JCPDS card no. 74-2184 and 41-1445, respectively. No other diffraction peaks resulting from other residues or impurity phases, e.g., ZnO, were found, indicating the successful synthesis of the as-obtained product. Notably, the ET-ZSO/SnO_2_ sample also had the same crystalline phases with P-ZSO/SnO_2_ even through the etching process, although the corresponding diffraction peaks became broader and the diffraction peak intensity of ET-ZSO/SnO_2_ grew weaker, compared with those of P-ZSO/SnO_2_. Other than ZSO and SnO_2_, no crystal phase related with Cu_2_O was detected in the ZSC1.5 sample, which might be attributed to the low concentration of Cu_2_O in comparison with that of ZSO and SnO_2_. On the other hand, pure Cu_2_O has sharp diffraction peaks, which are well indexed to JCPDS card no. 05-0667. Based on the analysis of XRD results, the possible etching mechanism for ZSO and SnO_2_ could be proposed as following. Being subject to a high concentrated solution of NaOH, Zn^2+^ and Sn^4+^ could respectively coordinate with a specified amount of OH^-^ to form soluble complex radicals of Zn(OH)_4_^2−^ and Sn(OH)_6_^2−^ under hydrothermal condition. As a result, the components of ZSO and SnO_2_ were continuously converted into soluble complex radicals and dissolved into the solution with hydrothermal time prolonging. In fact, the mass of P-ZSO/SnO_2_ decreased to a certain degree after etching treatment. Thus, such an explanation can be completely supported by the result that there is little difference in crystal phase and morphological structure before and after the etching treatment.

**Figure 4:**
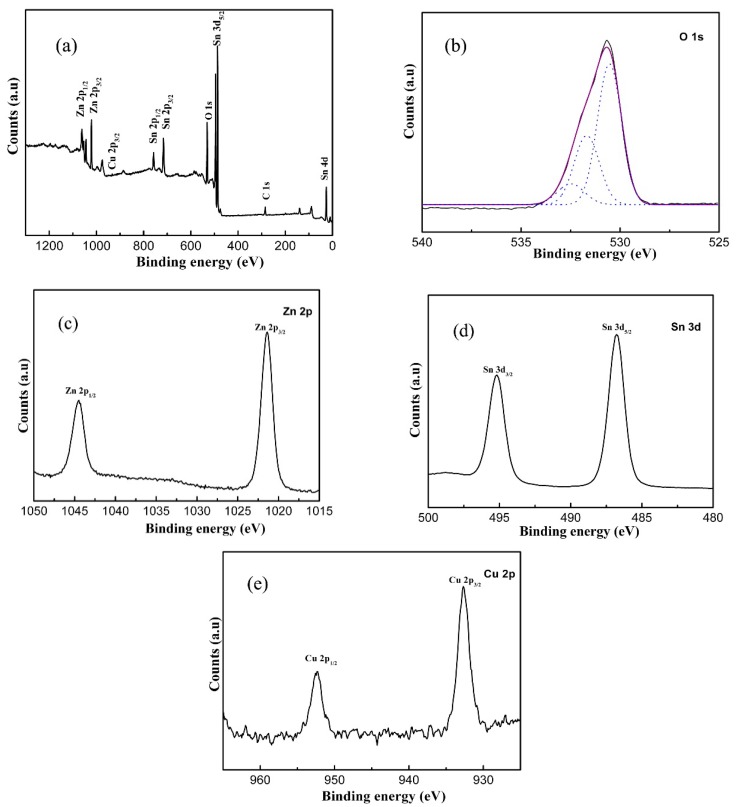
XPS spectra of the ZSC1.5 sample: (**a**) survey spectrum, (**b**) O 1s, (**c**) Zn 2p, (**d**) Sn 3d, (**e**) Cu 2p.

XPS analysis was taken to further inspect the surface component and valence status of the ZSC1.5 sample. The full scale XPS spectrum (Figure 4a) is constituted with four elements of O, Zn, Sn, and Cu, which is in good agreement with the result of EDS mapping. Referring to previously reported work, the asymmetric spectrum of O 1s (Figure 4b) can be divided into three peaks with binding energies at 530.5, 531.5 and 532.6 eV, which can be respectively denoted as lattice oxygen, vacancy oxygen, and chemisorbed oxygen species [17,36,37]. The vacancy oxygen species play a crucial effect on the visible-light photo-activity, as they are capable of inducing the formation of shallow energy levels. In terms of Zn 2p spectrum (Figure 4c), two fitting peaks with bind energies at ~1044.6 and 1022.4 eV could be ascribed to Zn 2p1_/2_ and Zn 2p_3/2_, respectively [15,17]. The prominent peaks centred at ~495.2 and 486.8 eV in Figure 4d can be assigned to the Sn 3d_1/2_ and Sn 3d _3/2_ of Sn^4+^ valence state. As for the chemical status of Cu element, the high-resolution spectrum of Cu 2p displayed two characteristic peaks with binding energies at ~952.4 and 932.7 eV, which verified the existence of Cu_2_O in the ZSC1.5 sample. Moreover, by calculation, the concentration of Cu_2_O is 1.4 wt%, being approximate to the theoretical value.

**Figure 5:**
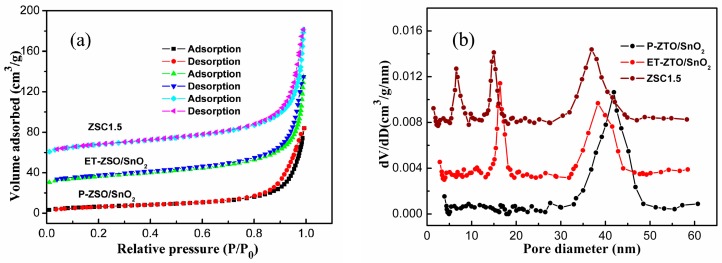
(**a**) The Nitrogen adsorption–desorption isotherm and (**b**) the corresponding pore size and distribution of P-ZSO/SnO_2_, ET-ZSO/SnO_2_ and ZSC1.5 samples.

It is common knowledge that photocatalytic redox reactions occur on the interface between photocatalysts and pollutants. As one of the most significant factors affecting the photo-activity of photocatalysts, absorption capacity is highly dependent on the surface specific area. Generally speaking, a photocatalyst with a larger surface area is likely to adsorb more pollutant molecules, being indicative of its excellent absorption capacity. As a consequence, the specific surface area, together with the porosity of the as-obtained products, was evaluated through nitrogen adsorption-desorption measurements. It is unambiguous from Figure 5a that three samples all displayed type IV isotherm accompanying with typical H3 hysteresis loop in the light of Brunauer-Deming-Deming-Teller (BDDT) classification, signifying the appearance of slit-like mesopores because of subunits stacking [16]. Consulting to the Brunauer–Emmett–Teller (BET) method, the specific surface area (S_BET_) of P-ZSO/SnO_2_, ET-ZSO/SnO_2_ and ZSC1.5 samples were approximately calculated to be 22.4 m^2^/g, 49.2 m^2^/g and 43.5 m^2^/g, respectively. By contrast, the S_BET_ value of ET-ZSO/SnO_2_ was much larger than that of P-ZSO/SnO_2_, signifying that the absorption capacity of ET-ZSO/SnO_2_ would be substantially enhanced due to the introduction of etching treatment. Although a slight decrease in the S_BET_ value occurred after Cu_2_O decoration, it is still rationally inferred that the ZSC1.5 sample will be provided with more active sites and exceptional absorption capacity as a novel photocatalyst, as compared with P-ZSO/SnO_2_. From Figure 5b, it is easily found out that all three samples contained mesopores ranging from 2nm to 50 nm. In comparison with P-ZSO/SnO_2_, some more mesopores with small sizes appeared for ET-ZSO/SnO_2_ and ZSC1.5, which was mainly attributed to the etching treatment and Cu_2_O modification. Such special characteristics of surface area and porosity would promisingly lead to high photo-activity for a multi-component photocatalyst.

**Figure 6:**
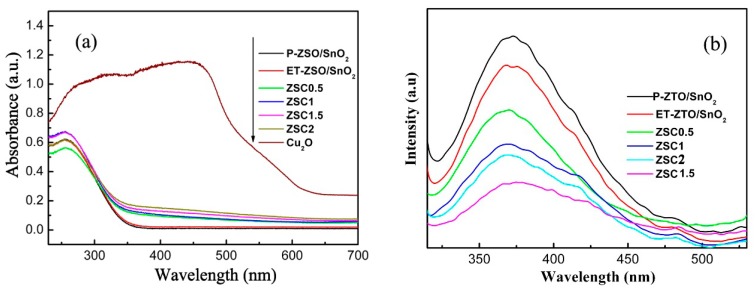
(**a**) UV–vis absorbance spectra of P-ZSO/SnO_2_, ET-ZSO/SnO_2_, and ZSC hybrids with different weight ratios, (**b**) PL spectra of P-ZSO/SnO_2_, ET-ZSO/SnO_2_, and ZSC hybrids with different weight ratios.

In general, different kinds of photocatalysts exhibit different absorption features, either in the light absorption intensity or light absorption range. For this purpose, the diffuse reflection spectra (DRS) of pristine P-ZSO/SnO_2_, ET-ZSO/SnO_2_, and ZSC samples with different weight contents were taken and the results are given in Figure 6a. Either P-ZSO/SnO_2_ or ET-ZSO/SnO_2_ presented a reasonably poor visible-light absorption property due to the wide band-gap of ZSO and SnO_2_. From another point of view, pure Cu_2_O displayed a wide light response range and outstanding light harvesting in the whole visible light region. After ET-ZSO/SnO_2_ are composited with Cu_2_O, all ZSC samples expectedly displayed evidently enhanced optical absorption in the whole visible light region, imputing to the interaction between fine Cu_2_O nanoparticles and ZSO and SnO_2_ nanorod assemblies.

As is well known, photoluminescence (PL) emission spectrum is confirmed to be feasible and effective for revealing the migration and recombination of photo-induced hole-electron pairs [6,38]. Specifically, a lower PL intensity signifies the lower recombination possibility of photogenerated hole-electron pairs. For this purpose, PL measurements for pristine P-ZSO/SnO_2_, ET-ZSO/SnO_2_, and ZSC hybrids were performed and the results are presented in Figure 6b. For P-ZSO/SnO_2_, the strongest intensity of PL emission peak was clearly indicative of the occurrence of the highest recombination possibility of photo-induced hole-electron pairs. With the addition of Cu_2_O, the PL emission peak intensity of ZSC hybrids declined sharply at first, then reached the lowest value, and tended to rise thereafter. ZSC1.5 exhibited the lowest PL emission intensity, revealing that it had the most efficient suppression of charge carrier recombination in this hybrid system. Moreover, it is worth noting that the emission peak with weaker intensity (centred at about ~480 nm) is probably ascribed to oxygen vacancies [39,40]. Based on the analysis above, wider photoresponse range and more efficient spatial separation of photogenerated charge carriers were simultaneously achieved for ZSC hybrids through the co-modification with etching treatment and Cu_2_O nanoparticle decoration.

**Figure 7:**
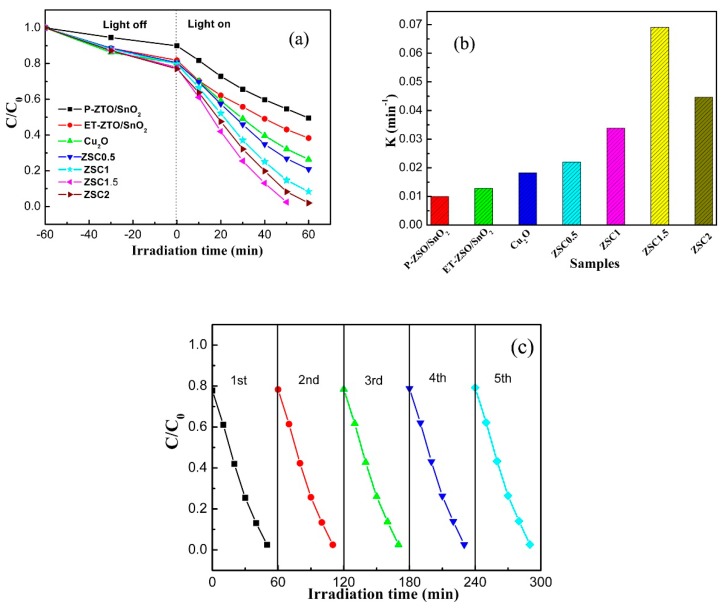
Photocatalytic degradation curves (**a**) and corresponding reaction kinetic constant curves (**b**) of P-ZSO/SnO_2_, ET-ZSO/SnO_2_, and ZSC hybrids with different weight ratios, (**c**) cycling runs in the photocatalytic degradation of RhB in the presence of ZSC1.5 sample.

In our work, the photocatalytic behaviour against the as-prepared photocatalysts was assessed with RhB as a target pollutant owing to its universal use and good long-term stability. For comparison, the photocatalytic degradation curve over M-ZSO is presented in Figure S2. Undoubtedly, the M-ZSO sample showed a very poor photocatalytic activity. Thus, it is particularly desired to carry out an overall investigation in order to explore the effect of the structural modulation and decoration on the photo-activity. As portrayed in Figure 7a, a certain amount of RhB molecules was adsorbed on the surface of photocatalysts during the absorption–desorption equilibrium process prior to illumination. By contrast, the adsorbed amount of RhB molecules for ET-ZSO/SnO_2_ and ZSC samples with different weight contents was larger than that for P-ZSO/SnO_2_, suggesting that the absorption capacity was indeed improved by the introduction of etching treatment, which was well consistent with the result of BET analysis. Under visible-light illumination, the absorbance values decreased continuously for all test samples. Among them, P-ZSO/SnO_2_ displayed the worst photodegradation efficiency, mainly owing to its characteristics of energy band and morphological structure. For ET-ZSO/SnO_2_ and ZSC samples, an evident improvement in photodegradation rate can be found, demonstrating that structural modulation combined with modification actually exerted a continuously positive effect on the photocatalytic performance. Specifically, the photodegradation efficiency of ZSC hybrids increased first with the Cu_2_O loading amount and reduced thereafter. The ZSC 1.5 sample exhibited the highest photodegradation rate, approaching 98% over a period of 50 min of visible-light illumination. Perhaps, excess Cu_2_O nanoparticles were likely to aggregate with each other and shadow the surface of ZSO and SnO_2_ from the incident visible-light photons, resulting in poor light exposure of ZSO and SnO_2_ for photo-induced charge carrier production. In order to quantitatively examine the reaction dynamics, we presumed that the photocatalytic behaviour for RhB aqueous solution followed the first-order kinetic model plotted by the equation of −ln(C/C_0_) = kt, in which k, C_0_ and C, respectively, stand for reaction rate constant, initial concentration, and concentration at time t of RhB concentration. From Figure 7b, it is very clear that ZSC1.5 had the highest kinetic constant of approximately 0.07 min^-1^, which was around 7 and 6 times higher than that of P-ZSO/SnO_2_ and ET-ZSO/SnO_2_. Considering that the usability and stability are also vital factors other than photocatalytic efficiency in the potentially practical application, ZSC1.5 was determined as the representative to recycle RhB degradation experiments for five runs, in which the photocatalyst was recovered by centrifugation, washing and drying at 65 °C for 10 h. The stability result for ZSC1.5 is given in Figure 7c. Only a slight decline was detectable in the photodegradation rate from the first to the fifth run, perhaps resulting from inevitable weight loss in the process of collecting and washing. The as-obtained results are indicative of high stability of ZSC hybrid photocatalyst.

**Figure 8:**
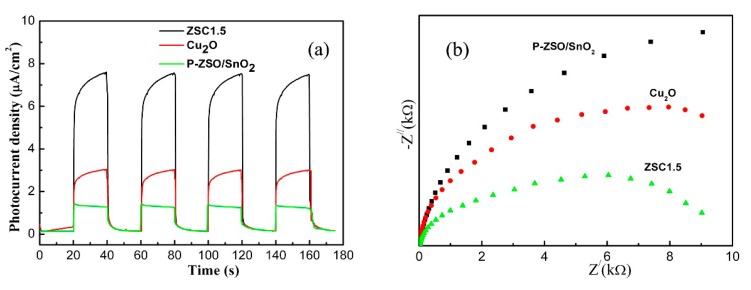
Transient photocurrents (**a**) and Electrochemical impedance spectra (**b**) of P-ZSO/SnO_2_, Cu_2_O, and ZSC hybrids electrodes under visible light irradiation (λ > 420 nm).

According to previously reported studies [41,42], the photocurrent response of photocatalysts can accurately shed light on photo-induced electrons and holes transfer and spatial separation. As a consequence, the photocurrent measurements of P-ZSO/SnO_2_, Cu_2_O and ZSC1.5 were carried out to further witness the contribution of Cu_2_O to the charge carrier spatial separation efficiency of ZSC hybrids. As shown in Figure 8a, ZSC1.5 presented the largest photocurrent among all the tested samples, suggesting that it owned the longest charge carrier lifespan and highest spatial separation efficiency. Furthermore, the capacity of interfacial charge separation was revealed by electrochemical impedance spectra (EIS) Nyquist plots of P-ZSO/SnO_2_, Cu_2_O and ZSC1.5 electrodes under visible-light illumination, as depicted in Figure 8b. Evidently, ZSC1.5 had a much smaller arc radius than P-ZSO/SnO_2_ and Cu_2_O, which was attributed to its smaller charge transfer resistance and faster interfacial transfer rate of photoinduced charge carriers [43,44,45].

**Figure 9:**
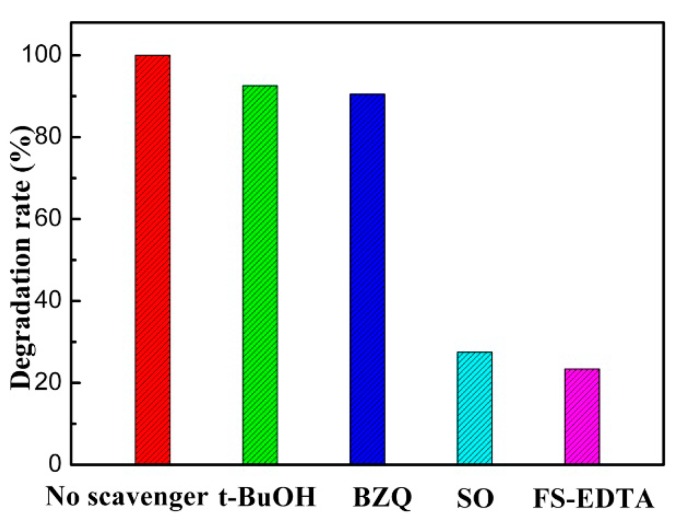
Effect of various scavengers on the visible-light photocatalytic performance of over the ZSC1.5 photocatalyst.

Trapping experiments were devised to reveal what active species would dominate the photodegradation reaction process in presence of ZSC1.5 photocatalyst. According to previous reports [46,47], four scavengers of benzoquinone (BZQ 2 mmol L^−1^), sodium oxalate (SO, 4 mmol L^−1^), tert-butyl-alcohol (t-BuOH, 2 mmol L^−1^), and FeSO_4_-EDTA (FS-EDTA, 0.2 mmol L^−1^) were individually put into the system to capture the active species of •O_2_^−^, h^+^, •OH and H_2_O_2_. The variation of photodegradation rate upon exposure to different scavengers is presented in Figure 9. Overall, the suppression effect on the photodegradation rate with the introduction of four scavengers followed the order of t-BuOH > BZQ > SO > FS-EDTA. Specifically, the photodegradation rate of RhB over ZSC 1.5 was only suppressed to some extent upon exposure to BZQ or t-BuOH, suggesting that the contribution of •O_2_^−^ and •OH was subordinate in the decomposition process. By contrast, the addition of SO and FS-EDTA led to an obviously significant decline in the photodegradation rate, implying that the active species of h^+^ and H_2_O_2_ could make a predominant contribution to photocatalytic oxidation reaction of RhB.

**Figure 10:**
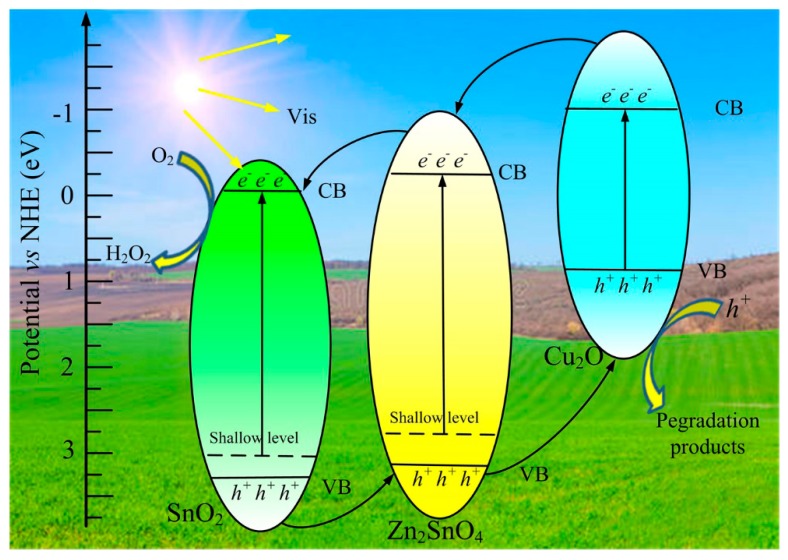
Schematic diagram of the charge transfer over the ZSC hybrids system under visible-light irradiation.

According to previous studies [15,21], the conduction band (CB) potentials for pure Cu_2_O, Zn_2_SnO_4_, and SnO_2_ are determined to be approximately −1.04 eV, −0.22 eV and 0.05 eV, respectively; while the valence band (VB) potentials for pure Cu_2_O, Zn_2_SnO_4_, and SnO_2_ are approximately 0.87 eV, 3.18 V and 3.25 eV, respectively. A proposed mechanism for the enormously promoted photocatalytic behavior is brought forth on the basis of energy-band structure combining with experimental results, as portrayed in Figure 10. In principle, pure Zn_2_SnO_4_ and SnO_2_ belong to the group of wide bandgap semi-conducted photocatalysts, and are incapable of being excited to generate electron-hole pairs under visible-light irradiation. As is revealed by aforementioned results of XPS and PL measurements, some defects, such as oxygen vacancy, existed in ZSC hybrid photocatalyst. As a result, shallow energy levels could be simultaneously produced in Zn_2_SnO_4_ and SnO_2_ photocatalysts [14,15,48]. Upon exposure to visible light illumination, the electrons from show energy levels of Zn_2_SnO_4_ and SnO_2_ are able to be excited and transfer to the CB positions of Zn_2_SnO_4_ and SnO_2_, respectively. As for narrow-bandgap Cu_2_O, the electrons from the VB position are easily excited, and then accumulate at the CB position through migration. Based on the energy band structure analysis above, a typical type II heterostructure is successfully established in the ternary component photocatalyst of Cu_2_O, Zn_2_SnO_4_ and SnO_2_. Assisted by the built-in electric field, the photoinduced electrons on the CB of Cu_2_O could flow to that of Zn_2_SnO_4_, eventually arrive, and accumulate at the CB position of SnO_2_. Simultaneously, the photoinduced holes from VB of SnO_2_ are available to accumulate at the VB position of Cu_2_O against the direction of the electron flow. Therefore, the efficient separation of photogenerated electron-hole pairs is successfully achieved in the ZSC hybrid system composed of Cu_2_O, Zn_2_SnO_4_, and SnO_2_ components with a well-matched band structure. It is worthwhile to note that the CB potential of SnO_2_ (0.22 eV) is negative enough to enable electrons to react with O_2_ and to generate the species of H_2_O_2_ (O_2_/H_2_O_2_, 0.695 eV) [49,50,51], significantly playing a positive role in the photocatalytic oxidation process of RhB molecules. Although the VB potential of Cu_2_O (0.87 eV) is not oxidative enough to convert the radicals of OH^-^ into the active species of OH (OH^−^/•OH, 1.99 eV) [52,53,54,55], the accumulated holes on the VB of Cu_2_O could straightforwardly oxidize organic pollutant molecular to CO_2_ and H_2_O. That is why the active species of h^+^ and H_2_O_2_ exerted a predominant contribution to the oxidization process of organic pollutant molecular.

## 4. Conclusions

In summary, Zn_2_SnO_4_/SnO_2_/Cu_2_O hybrid photocatalysts were successfully achieved via a novel “directing growth-etching treatment-coupling composites” route based on structural modulation and band alignment modification. The co-modification of the etching treatment and Cu_2_O decoration endowed ZSC hybrid photocatalysts with a larger specific surface area, better light absorption ability, and more efficient spatial separation of photogenerated charge carriers. Thus, the resultant ZSC hybrid photocatalyst exhibited a continuously improved photocatalytic performance towards the photodegradation of RhB, as compared with the P-ZSO/SnO_2_ sample. Especially, the k value of the ZSC1.5 photocatalyst reaches up to 0.07 min^−1^, which was, respectively, about seven times higher than the corresponding counterparts for the P-ZSO/SnO_2_ sample. Such a simply and rationally designed route can provide an idea for the synthesis of highly efficient visible-light responsive photocatalysts applied in the territory of solar energy utilization and conversion.

## Supplementary Materials

The following are available online at https://www.mdpi.com/2079-4991/9/10/1390/s1, Figure S1: SEM image of Zn_2_SnO_4_/SnO_2_ microspheres, Figure S2: Photocatalytic degradation curve of Zn_2_SnO_4_/SnO_2_ microspheres.
