# Supplementary material for: Continuously Improved Photocatalytic Performance of Zn2SnO4/SnO2/Cu2O Composites by Structural Modulation and Band Alignment Modification
Source: Nanomaterials (Basel). 2019 Sep 28;9(10):1390. doi: 10.3390/nano9101390 (PMC6835881; doi:10.3390/nano9101390)
Supplement: Supplementary file 1 [file nanomaterials-09-01390-s001.pdf]

Supporting Information

Continuously Improved Photocatalytic Performance of $Zn_2SnO_4/SnO_2/Cu_2O$ Composites by Structural Modulation and Band Alignment Modification

Tiekun Jia [1,*], Junchao An [1,*], Dongsheng Yu [1], Jili Li [1], Fang Fu [1], Kun Wang [1] and Weimin Wang [2]

[1] School of Materials Science and Engineering, Luoyang Institute of Science and Technology, Wangcheng Road 90#, Luoyang 471023, China
[2] State Key Lab of Materials Synthesis and Processing, Wuhan University of Technology, Wuhan 430070, China
* Correspondence: tiekunjia@126.com or tkjia@whut.edu.cn (T.J.); superjun@tju.edu.cn (J.A.); Tel./Fax: +86-379-65928196 (T.J.)

Received: 24 August 2019; Accepted: 21 September 2019; Published: date

1. Experimental section (Synthesis of Zn_2SnO_4/SnO_2 microspheres)

In a typical process, 1 mmol of $SnCl_4·5H_2O$ was dissolved in ethanol (20 mL) with continuous stirring for 20 min to yield a homogeneous solution (marked as solution A). Meanwhile, 1 mmol of $Zn(CH_3COO)_2·2H_2O$ was dissolved in deionized water (20 mL) to achieve a transparent solution (marked as solution B). Subsequently, solution A was added into solution B with continuous stirring. After that, NaOH aqueous solution (10 mL 2 M) was slowly dripped into the aforementioned mixed solution under vigorous stirring. The product was collected by centrifugation and washed with deionized water and ethanol several times, followed by drying at 70 °C in an electric oven overnight. Subsequently, the resulting product was annealed at 600 °C in air for 2 h to obtain Zn_2SnO_4/SnO_2 microspheres.

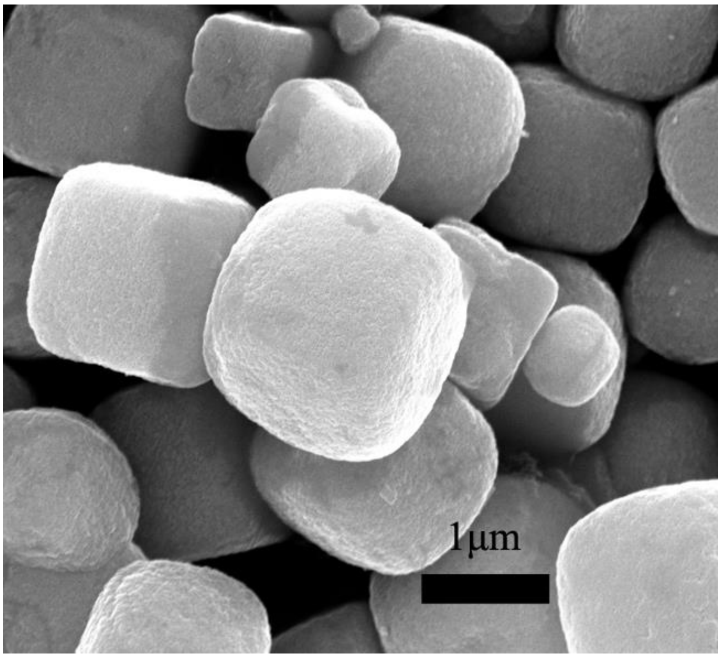

Figure S1. SEM image of Zn_2SnO_4/SnO_2 microspheres.

2. Photocatalytic performance

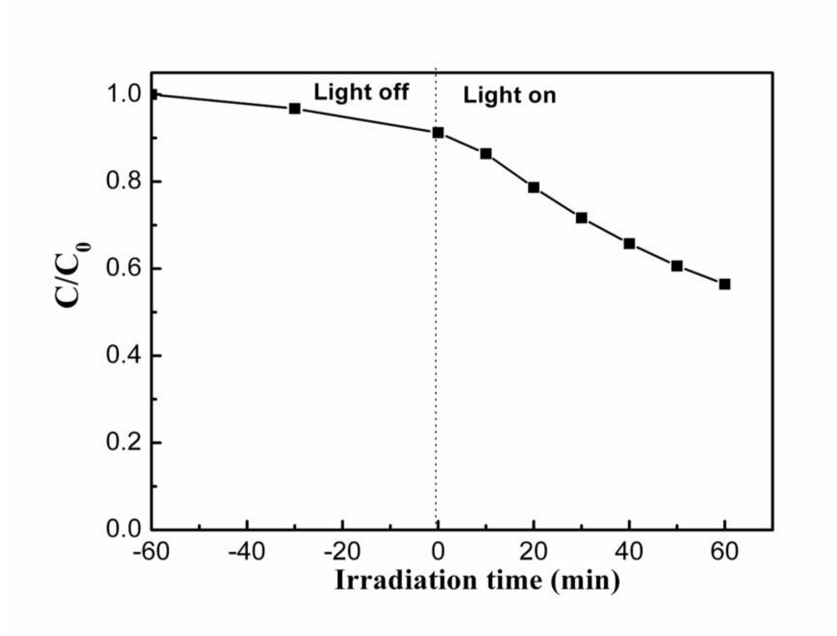

Figure S2. Photocatalytic degradation curve of Zn_2SnO_4/SnO_2 microspheres.
